# A cobalt concentration sensitive Btu-like system facilitates cobalamin uptake in *Anabaena* sp. PCC 7120

**DOI:** 10.15698/mic2024.02.814

**Published:** 2024-02-20

**Authors:** Julia Graf, Leonard Fresenborg, Hans-Michael Seitz, Rafael Pernil, Enrico Schleiff

**Affiliations:** 1 Institute for Molecular Biosciences, Goethe University Frankfurt, Max von Laue Str. 9, 60438 Frankfurt, Germany.; 2 Frankfurt Isotope and Element Research Center, Goethe University Frankfurt, 60438 Frankfurt Germany.; 3 Institute for Geoscience, Goethe University Frankfurt, Altenhöferallee 1, 60438 Frankfurt, Germany.; 4 Frankfurt Institute for Advanced Studies, Ruth-Moufang-Straβe 1, 60438 Frankfurt, Germany.; 5 Buchmann Institute for Molecular Life Sciences, Max von Laue Str. 11, 60438 Frankfurt, Germany.

**Keywords:** cyanobacteria, cobalt, cobalamin, riboswitch, metal uptake, TonB-dependent transporter

## Abstract

Metal homeostasis is central to all forms of life, as metals are essential micronutrients with toxic effects at elevated levels. Macromolecular machines facilitate metal uptake into the cells and their intracellular level is regulated by multiple means, which can involve RNA elements and proteinaceous components. While the general principles and components for uptake and cellular content regulation of, e.g., cobalt have been identified for proteobacteria, the corresponding mechanism in other Gram-negative bacteria such as cyanobacteria remain to be established. Based on their photosynthetic activity, cyanobacteria are known to exhibit a special metal demand in comparison to other bacteria. Here, the regulation by cobalt and cobalamin as well as their uptake is described for *Anabaena* sp. PCC 7120, a model filamentous heterocyst-forming cyanobacterium. *Anabaena* contains at least three cobalamin riboswitches in its genome, for one of which the functionality is confirmed here. Moreover, two outer membrane-localized cobalamin TonB-dependent transporters, namely BtuB1 and BtuB2, were identified. BtuB2 is important for fast uptake of cobalamin under conditions with low external cobalt, whereas BtuB1 appears to function in cobalamin uptake under conditions of sufficient cobalt supply. While the general function is comparable, the specific function of the two genes differs and mutants thereof show distinct phenotypes. The uptake of cobalamin depends further on the TonB and a BtuFCD machinery, as mutants of *tonB3 and btuD* show reduced cobalamin uptake rates. Thus, our results provide novel information on the uptake of cobalamin and the regulation of the cellular cobalt content in cyanobacteria.

## INTRODUCTION

Cyanobacteria by the photosynthetic nature are essential for global CO_2_ fixation and O_2_ production. Their growth and development is thus dependent on micronutrients. These vary in their bioavailability depending on the environment cyanobacteria life in and on changes thereof. In response to this, different uptake systems and pathways have evolved and their components are encoded in the genomes of cyanobacteria. The transport of solutes involves, for example, porins for salt and other nutrients (e.g., [1–3]). Solutes that exceed the diffusion size of porins or have a very low abundance in the environment are actively transported by specialized systems (e.g., [4]). Outer membrane TonB-dependent transporters (TBDTs; [5]) are one family involved in such processes. They are 22-stranded β-barrel proteins with an N-terminal globular plug domain and a TonB box (TonBB) that is recognized by the TonB protein in the plasma membrane [6]. Although TBDTs share a certain degree of structural conservation, the individual TBDTs are specialized for different substrates [7], which explains the existence of multiple TBDTs in the outer membrane of a single organism.

The TonB protein in complex with ExbB/D provides the energy for the transport of iron-loaded siderophores across the outer membrane by converting the electrochemical gradient across the plasma membrane [6, 8]. After substrate binding to the TBDT, conformational changes catalyze the substrate transport into the periplasm where periplasmic binding proteins capture it. The transport across the plasma membrane of bacteria depends on ABC-type uptake transporters that consist of a periplasmic binding protein, two transmembrane permeases, and two ATPase proteins located in the cytoplasmic face of the plasma membrane [9].

*Anabaena* sp. PCC 7120 (hereafter *Anabaena*) is a model organism for Gram-negative filamentous cyanobacteria with the ability to produce heterocysts under nitrogen deprivation (e.g. [10, 11]). Each cell in a filament is individually surrounded by a plasma membrane and a peptidoglycan layer, while the entire filament is enclosed by a continuous outer membrane [12, 13]. The latter contains multiple protein families to ensure biogenesis and transport [14]. In the genome of *Anabaena* 22 genes code for putative TBDTs [15, 16]. Four of them have been characterized, namely the iron and copper transporter IacT and three TBDTs responsible for the uptake of schizokinen (SchT, IutA1, IutA2; [17–20]). The functional identity of the other 18 TBDTs has been predicted, but experimental confirmation is still missing. This holds true for the gene products of *all3310* (*btuB1*) and *alr4028-alr4029* (*btuB2; alr4028-alr4029* constitute a single gene because the stop codon in between was a genome annotation error) [15].

**Figure 1:**
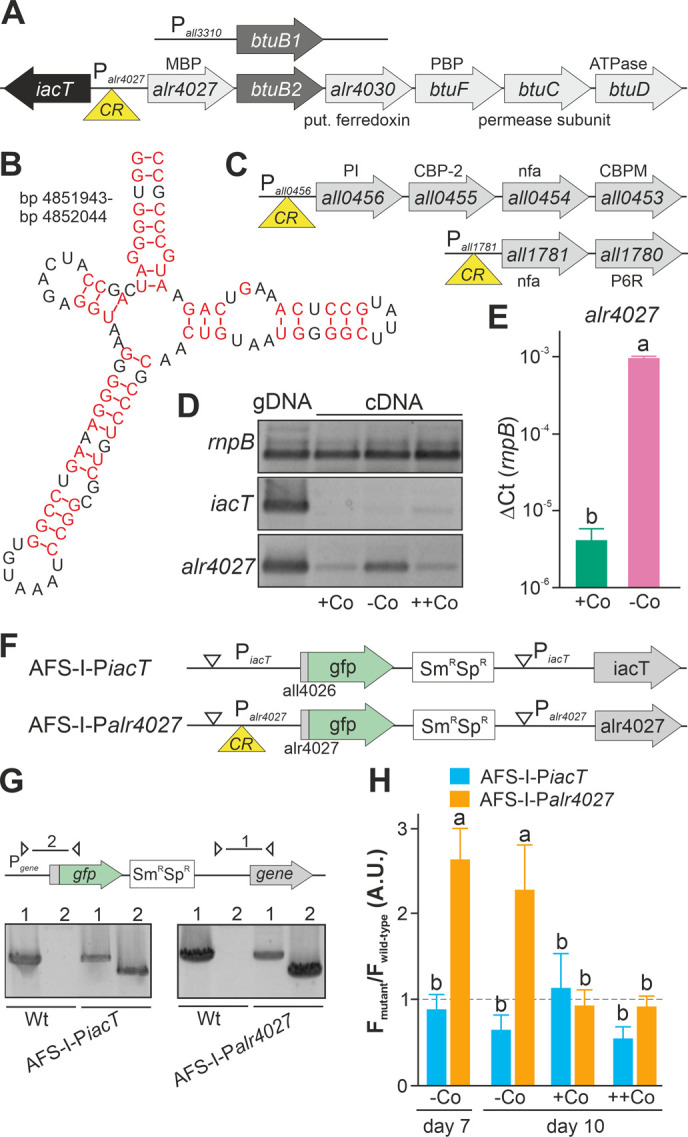
FIGURE 1. Regulation of translation by a cobalamin riboswitch. **(A)** Genomic organization of the region containing *btuB1* and *btuB2*. Yellow triangle marks the riboswitch position. CR: cyanocobalamin riboswitch; MBP: metal-binding protein; PBP: periplasmic binding protein. **(B)** Predicted structure of the adenosylcobalamin riboswitch encoded in the 5' region of *alr4027*. **(C)** Genomic organization of two gene clusters with identified cyanocobalamin riboswitch. CBP-2: cobalamin biosynthesis precorrin-2; CBPM: cobalamin biosynthesis precorrin-3 methylase; nfa: no functional assignment; P6R: precorrin-6x reductase; PI: precorrin isomerase. **(D)** gDNA (lane 1) and cDNA (lane 2-4) of *Anabaena* grown for 14 days in YBG11 medium without cobalt and subsequently grown for seven days in YBG11 (+Co, lane 2), in YBG11 without cobalt (-Co, lane 1 and 3) or YBG11 with 5 µM CoCl_2_ (++Co, lane 4) media was analyzed by PCR using oligonucleotides for the indicated genes. **(E)** The cDNA (+Co, -Co) used in D was analyzed by qPCR and values were normalized to the level of *rnpB*. **(F)** Model of the strategy for genomic plasmid insertion to generate AFS-I-P*iacT* and AFS-I-P*alr4027.* **(G)** Genomic DNA isolated from wild-type *Anabaena*, AFS-I-P*iacT* and AFS-I-P*alr4027* was used for PCR with gene-specific primers (lane 1) or a plasmid- and a gene-specific primer (lane 2) as indicated in the model on top. **(H)** Wild-type *Anabaena*, AFS-I-P*iacT* (blue) and AFS-I-P*alr4027* (orange) were grown in YBG11 medium without cobalt for seven (first bar) or ten days (second bar). After seven days the culture was transferred into a new medium for growth for three days in YBG11 (+Co, third bar) or in YBG11 with 5 µM CoCl_2_ (++Co, fourth bar) media. The ratio of the GFP fluorescence of the mutant strain and the background in wild-type *Anabaena* is shown and the background level is indicated as a dashed line. In (E) and (H) the results represent the average of at least three independent experiments; error bars show standard deviation and letters indicate the ranks based on statistical analysis performed using ANOVA (p<0.05).

In *Escherichia coli*, BtuB-like TBDTs together with the periplasmic binding protein BtuF and the ABC-type transporter composed of BtuC and BtuD transport various cobalamins [21, 22]. In *Anabaena btuB1* stands alone in the genome, while *btuB2* (*alr4028-alr4029*) is part of a gene cluster that also contains *alr4031* (periplasmic binding protein), *alr4032* (permease subunit) and *alr4033* (ATPase; **Figure 1A**; [15]). In agreement with the nomenclature established for the Btu system in *E. coli* (e.g. [21]), we propose to annotate *alr4031* as *btuF*, *alr4032* as *btuC* and *alr4033* as *btuD*. BtuE was previously characterized as a glutathione peroxidase in *E. coli* [23]. As for the protein encoded by *alr4030* a similarity to ferredoxins was proposed [15], we did not annotate this gene as *btuE*.

The terms cobalamin and pseudocobalamin both refer to compounds that contain a corrin macrocycle structure (corrinoids) chelating a central cobalt ion (cobamides, [24]). In contrast to cobalamin, defined as the cobamides with physiological activity in humans, in pseudocobalamins the lower axial ligand 5,6-dimethyl-benzimidazole (DMB) is replaced with an adenine moiety. They differ in the upper and lower axial ligands bound to the cobalt ion [21]. BtuB from *Vibrio cholerae* can transport both cobalamins and pseudocobalamins [25] and *E. coli* BtuB was successfully hijacked for import of cyanocobalamin-modified peptide nucleic acids [26]. In agreement with those findings, crystallography revealed little interaction between the BtuB substrate binding loops and the axial ligands [27], strongly indicating that BtuB proteins are generic cobamide importers specific to the corrin ring. Whether BtuB proteins recognize corrinoids containing no metal ion or a different one remains unknown.

Both pseudocobalamin and cobalamin biosynthesis has been demonstrated in cyanobacteria, although several reports of cobalamin are from non-axenic material [28] or material without indication of the culture purity [22, 29–31]. Consistently, *Anabaena* has genes encoding proteins involved in the synthesis of such metabolites [22]. Cyanobacteria typically possess genes for cobamide biosynthesis but most lack genes for DMB biosynthesis [32]. Hence it is discussed that the group predominantly synthesizes pseudocobalamin, which is less bioavailable to microalgae than cobalamin [22, 32]. To the best of our knowledge there is not yet a report presenting direct experimental evidence for the production of pseudocobalamin by this particular cyanobacterial strain. Nevertheless, due to the presence of the genes for the synthesis we assume that *Anabaena* is able to produce pseudocobalamin or cobalamin, which needs to be experimentally confirmed in the future.

As the central ion of (pseudo)cobalamin [22], the cofactor of the methionine synthase [32–34], cobalt is essential for cyanobacteria as documented by starvation experiments [35–39]. Moreover, it is thought to have the ability to partially replace other micronutrients [40]. Its importance is consistent with a tight regulation of the synthesis and uptake system. Here, *btuB* genes are often under the regulatory control of a so-called cobalamin riboswitch. Cobalamin riboswitches vary in specificity with some preferring a specific cabamide, often adenosylcobalamin, and some binding corrinoids promiscuously [41]. For instance, evidence supports that methylcobalamin is a ligand of some cobalamin riboswitches [42–44]. In *E. coli* [45] and *Salmonella typhimurium* [46] cobalamin was found to reduce the translation of BtuB. Subsequently, it was demonstrated that adenosylcobalamin inhibits the RNA binding to ribosomes by physical interaction with the RNA [43, 44, 47]. Although the cobalamin riboswitch acts in regulation of translation, in *E. coli* the mRNA abundance of the gene under the control of the riboswitch is strongly correlated with a cobalamin-dependent regulation [48] and for *Synechococcus* sp. PCC 7002 a function of the cobalamin riboswitch as a transcription terminator was postulated [33].

For *btuB1* a transcriptional start site is found upstream of the gene [49] and for *btuB2* a transcriptional start site appears to be located within *all4026* [49]. This suggests the existence of a transcriptional unit of at least *alr4027* and *btuB2*, because a transcriptional start site was also found upstream of *alr4030* [49]. Remarkably, based on this analysis, the region *alr4030* to *btuD* appears to represent a transcriptional unit as well. Insertional mutants of *btuB1* and *btuB2* were not affected in Fe-schizokinen uptake [20], but transport of cobalamin has not been explored so far. Moreover, *btuB1* was found to be transcribed at basal levels under many different growth conditions [15, 50]. In contrast, *btuB2* was only found to be transcribed under elevated iron or copper levels [15, 50].

In this article, we demonstrate that the *btuB*-like gene *btuB2* and the ABC-type transport system encoded immediately downstream (*btuFCD*) are both induced by Co-deprivation and facilitate the uptake of cyanocobalamin in *Anabaena*. Furthermore, we provide evidence that the starvation-specific expression of the genes *alr4027*-*btuD* occurs in the form of a single polycistronic transcript and depends on a genetic element upstream of *btuB2*.

## RESULTS AND DISCUSSION

### Cobalamin riboswitch is sensitive to cobalt starvation

Upstream of *alr4028*, located between *iacT* (*all4026*, **Figure 1A**; [17]) and *alr4027* (putative metal-binding protein, [15]), a sequence with similarity to a cobalamin riboswitch (E=1.3*10^-10^) with slightly higher similarity to an adenosylcobalamin riboswitch was found (E-value 3*10^−13^; **Figure 1B**). A global analysis of the genome uncovered additional putative cobalamin riboswitches in the 5' region of *all1781*, which together with *all1780* encodes proteins for vitamin B_12_ synthesis, and in the 5' region of *all0456*, which is the first gene of a cluster encoding factors for vitamin B_12_ synthesis (**Figure 1C**). This suggests that the intracellular cobalt abundance regulates the uptake and synthesis of cobalamin.

The dependence of the transcription of *alr4027* and *iacT* on exogenous cobalt availability was analyzed. Wild-type *Anabaena* was grown in the absence of cobalt for two weeks and subsequently subjected to media containing a standard amount of cobalt (**Figure 1D**, +Co), no cobalt (-Co) or excess of CoCl_2_ (++Co) for seven days. Prior to the latter experiment, the toxicity level of cobalt was tested by growth of wild-type *Anabaena* in the presence of different concentrations of CoCl_2_. An IC_50_ value of 18 µM CoCl_2_ was observed with 95% toxicity in the presence of 25 µM CoCl_2_. The growth medium YBG11 contains 0.2 µM CoCl_2_ and YBG11 with enhanced cobalt a concentration of 5 µM CoCl_2_.

Expression of *iacT* was only observed at low level in the presence of a high amount of cobalt (middle panel, last lane), whereas *alr4027* was expressed under all conditions but at the highest level under prolonged cobalt starvation (lower panel). The expression of *rnpB* was not affected by the treatments. qPCR on cDNA indicated that *alr4027* transcript level under -Co conditions was about 100-fold higher when compared to normal YBG11 medium (+Co; **Figure 1E**). Thus, the transcript abundance of *alr4027* is regulated by cobalt availability.

As orthologues of the putative cobalamin riboswitch are involved in translational regulation [45, 46], we decided to verify our findings with a reporter gene assay to detect possible post transcriptional effects. Translational fusions of the green fluorescent protein (GFP) to the promoter regions of *iacT* and *alr4027* were generated to probe the influence of exogenous cobalt on the translation of both proteins (**Figure 1F**). The fusions contain the start codon of the respective gene and are under the control of the native promoters. The constructs were transferred into wild-type *Anabaena* by conjugation and insertion into the genome was confirmed by colony PCR (**Figure 1G**). The generated mutants, AFS-I-P_*iacT*_ and AFS-I-P_*alr4027*_, as well as the wild-type *Anabaena* were subjected to cobalt starvation for seven days and the GFP fluorescence was monitored (**Figure 1H**, bar 1). As observed at RNA level, IacT translation was not induced by cobalt starvation as the GFP fluorescence of the corresponding mutant was at a background level similar to that observed for the wild type. In contrast, translation of Alr4027 was induced by cobalt starvation. Subsequently, cells were transferred into YBG11 without cobalt, standard YBG11 or YBG11 with excess of CoCl_2_ media and GFP fluorescence was measured again after three days (**Figure 1H**, day 10). GFP production was not observed in AFS-I-P_*iacT*_ irrespective of the treatment as the signal remained at background level (**Figure 1H**, -Co, +Co, ++Co). Conclusively, cobalt presence or absence does not affect *iacT* expression. In AFS-I-P_*alr4027*_ the GFP level remained high during prolonged starvation (**Figure 1H**, -Co, day 7 vs. day 10). In the presence of cobalt, the GFP production was suppressed to background level as the GFP fluorescence level was similar as in the wild-type (**Figure 1H**, +Co, ++Co). Considering the remaining basal expression of *alr4027* in the presence of cobalt (**Figure 1D**), these results confirm that the transcription/translation of *alr4027* is regulated by cobalt abundance but the expression of *iacT* is not. As the regulatory pattern is qualitatively identical to that found by RT-PCR, we conclude that the observed regulatory effect is either caused solely by transcriptional regulation or by factors that cooperatively act on both mRNA and protein biosynthesis. Based on the empirically observed Co-dependent regulation and the similarity to genetic arrangements investigated in other bacteria, we suggest the following hypothesis: The gene *alr4027* is transcribed from a promotor within *iacT*. Hence, it is co-transcribed with the putative Co-dependent riboswitch encoded in the intergenic region (**Figure 1A, B**). However, considering the low expression level of *alr4027* under cobalt-replete conditions, it seems possible that an additional transcriptional start site may exist which was not identified by [49].

### Cobalt starvation induces *btuB2,* but not *btuB1*

**Figure 2:**
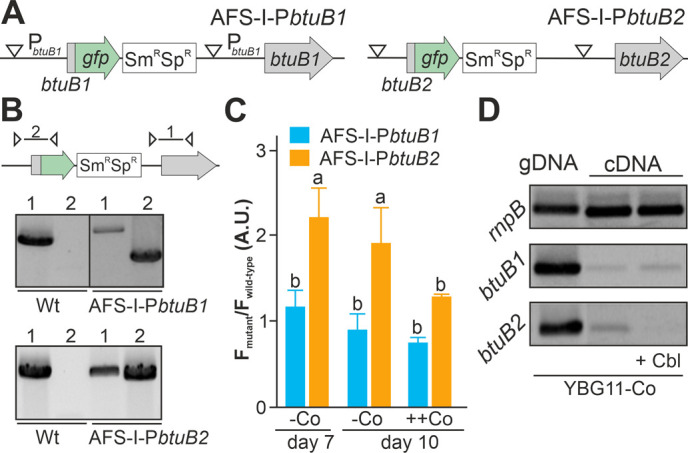
FIGURE 2: Regulation of expression of *btuB1* and *btuB2*. **(A)** Model of the genomic plasmid insertion to generate AFS-I-P*btuB1* and AFS-I-P*btuB2.* White triangle marks the position of the predicted promoter start. **(B)** Genomic DNA isolated from wild-type *Anabaena*, AFS-I-P*btuB1* and AFS-I-P*btuB2* was used for PCR with gene-specific (lane 1) or a plasmid- and a gene-specific primer pair (lane 2) as indicated in the model on top. **(C)** Wild-type *Anabaena*, AFS-I-P*btuB1* (blue) and AFS-I-P*btuB2* (orange) were grown in YBG11 medium without cobalt for seven (first bar) or ten days (second bar). After seven days the culture was transferred for growth for three days in YBG11 medium with 5 µM CoCl_2_ (++Co, third bar). The ratio of the GFP fluorescence of the mutant strain and the background in wild-type *Anabaena* is shown. **(D)** gDNA (lane 1) and cDNA (lane 2 and 3) of *Anabaena* grown for 14 days in YBG11 medium without cobalt and subsequently grown for seven days in YBG11 without cobalt (lane 1 and 2), but with 5 µM cobalamin (+Cbl, lane 3) media was analyzed by PCR using oligonucleotides for the indicated genes. In (C) the average of three independent experiments is shown; error bars indicate standard deviations and the letters indicate the ranks based on statistical analysis performed using ANOVA (p<0.05).

As the non-coding region upstream of *btuB1* lacks a putative riboswitch, Co-dependent regulation of the gene would have to result from a different genetic mechanism. In contrast to that, *btuB2* would be expected to be co-regulated with *alr4027*, assuming they are located on the same polycistronic mRNA as suggested [15, 49]. To test that argument, the translation of *butB1* and *btuB2* was analyzed using the translational GFP fusions (**Figure 2A**). The genome insertion of AFS-I-P_*btuB1*_ and AFS-I-P_*btuB2*_ was confirmed (**Figure 2B**), but we did not probe chromosome segregation, as wild-type chromosomes were not expected to have impact on the experiment. As before, GFP fluorescence was measured in wild-type (background) and mutant strains grown for seven or ten days in the absence of cobalt (**Figure 2C**, -Co, day 7 and day 10). No GFP signal above background level was detected while analyzing AFS-I-P_*btuB1*_, irrespective of the medium used. Consistent with our previous results for AFS-I-P_*alr4027*_, GFP fluorescence was observed in AFS-I-P_*btuB2*_ after seven or ten days of cobalt starvation (**Figure 2C**, -Co). When cells after seven days of starvation were incubated in YBG11 medium with excess of cobalt (5 µM CoCl_2_), the GFP-fluorescence of the strain AFS-I-P_*btuB2*_ was reduced to background level (**Figure 2C**, ++Co).

Inspired by the observation that the cobalamin riboswitch, although primarily regulating translation, has an impact on mRNA stability [33, 48], the existence of such relation in *Anabaena* was analyzed. The transcriptional level of *btuB1* and *btuB2* was probed by RT-PCR on mRNA isolated from wild-type grown under cobalt starvation conditions for 14 days followed by transfer to YBG11 without cobalt (**Figure 2D**, lane 2) or YBG11 without cobalt but with 5 µM cobalamin (**Figure 2D**, lane 3). While *btuB1* is transcribed at low level irrespective of the treatment (panel 2), *btuB2* transcripts are clearly detectable under cobalt starvation conditions (panel 3, lane 2), but absent after addition of cobalamin (panel 3, lane 3).

In summary, *btuB2* or *GFP* replacing *btuB2* exhibit the same Co-dependent regulatory pattern as the preceding *alr4027*, whereas *btuB1*, which lacks the putative riboswitch, is expressed Co-independently. Similarly, mRNA abundance of *btuB2* and *alr4027* appears to be cobalt sensitive, whereas the transcript abundance of *btuB1* is not. The conclusion is that the riboswitch upstream of *alr4027* is the element that confers a Co-dependent regulation.

### Impact of *btuB* loss-of-function mutants on expression of *btuB1*, *btuB2* and *btuC*

**Figure 3:**
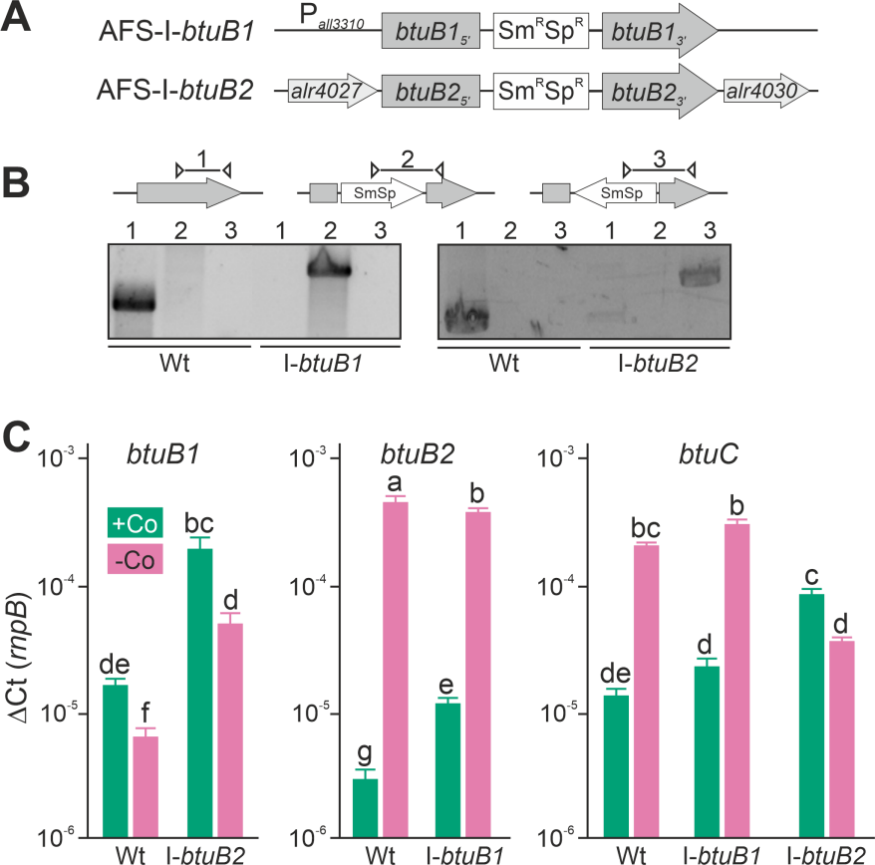
FIGURE 3: Expression of *btuB1, btuB2* and *btuC* in *btuB* loss-of-function mutants. **(A)** Model of the strategy for genomic plasmid insertion to generate AFS-I-*btuB1* and AFS-I-*btuB2.* **(B)** Genomic DNA isolated from wild-type *Anabaena* and mutant strains AFS-I-*btuB1* and AFS-I-*btuB2* was used for PCR with gene-specific primers (lane 1), a gene-specific primer and a plasmid-specific 3' (lane 2) or 5' primer (lane 3). **(C)** The cDNA of the indicated strains grown in the absence of cobalt for 14 days and subsequently in the presence or absence of cobalt (+Co, green; -Co, magenta) for seven days was analyzed by qPCR and values were normalized to the expression level of *rnpB*. In (C) the average of three independent experiments is shown; error bars indicate standard deviations and the letters indicate the ranks based on statistical analysis performed using ANOVA (p<0.05).

Plasmid-insertion mutants in *btuB1* and *btuB2* were created by single recombination (**Figure 3A**) to further explore the interdependence of *btuB1* and *btuB2* expression. All mutants were segregated as a product with the gene-specific oligonucleotides could not be detected (**Figure 3B**, lane 1), while the plasmid insertion could be confirmed with a plasmid- and a gene-specific oligonucleotide primer (**Figure 3B**, lane 2 or 3). The strains were annotated as AFS-I-*btuB1* and AFS-I-*btuB2* (**Figure 3A**).

First, we searched for polar effects of the insertions on neighboring genes and other forms of expression interdependence. To that end, wild-type *Anabaena* and both mutants were grown in YBG11 medium without cobalt for 14 days, followed by seven days of incubation in YBG11 medium without cobalt or YBG11 medium. Subsequently, mRNA was isolated and used for RT-qPCR.

In the wild-type, the results confirmed those found with end-point PCR (**Figure 2D**). The transcription of *btuB1* was detectable under both conditions, but not enhanced by cobalt starvation (**Figure 3C**, left graph, bar 1 and 2). In fact, the expression level was found to be significantly lower under cobalt depletion. In contrast, the transcript of *btuB2* was rather of low abundance in the presence of cobalt (middle graph, bar 1) but highly upregulated (100-fold) when exposed to cobalt starvation (middle graph, bar 2). In addition, the transcript abundance of *btuC* was close to that of *butB1* in the presence of cobalt, and was at least ten-fold increased under cobalt starvation (**Figure 3C**, right graph, bar 1 and 2). Thus, *btuC* expression behaves qualitatively identically to that of *alr4027* (**Figure 1D, E, H**) and *btuB2* (**Figure 2C**, **Figure 3C**). This suggests that *btuC* is included in the putative polycistronic mRNA that contains *alr4027* and *btuB2*. In agreement, the relative gene expression values of *alr4027* (**Figure 1E**), *btuB2* and *btuC* (**Figure 3C**) decrease under cobalt starvation in the direction of translation as it would be expected for a polycistronic arrangement. While *alr4027* and *btuB2* have the same expression level in cobalt-replete medium (**Figure 1E**, **Figure 3C**), the expression of *btuC* in this medium is about five times higher compared to these two genes. A possible explanation for this discrepancy is the additional transcriptional start site identified upstream of *alr4030*. An additional Co-independent transcript initiation at this site would increase the cobalt replete expression level of *btuF, btuC* and *btuD*. This suggests that the ABC-type import system is active under both, Co-replete and deplete conditions, while BtuB2 is specific to Co starvation.

Interestingly, in AFS-I-*btuB2* the expression of *btuB1* was ten-fold enhanced when compared to the wild-type, reaching a level similar to that of *btuB2,* irrespective of whether the strains were grown in the presence or absence of cobalt (**Figure 3C**, left graph, bar 1 vs. 3 and 2 vs. 4). This suggests a functional relationship between the two BtuB-like proteins. As for the wild-type, significantly lower expression of *btuB1* was found under Co-deplete conditions. Correspondingly, the loss of function of *btuB1* in AFS-I-*btuB1* resulted in a three-fold enhanced expression of *btuB2* in comparison to the wild-type when the strains were grown in the presence of cobalt (middle graph, bar 1 vs. 3). However, in the absence of cobalt, no additional increase of *btuB2* expression was observed in the mutant strain (middle graph, bar 2 vs. 4). This suggests that *btuB2* in part compensates for the loss of *btuB1* under normal growth conditions. The absence of an additional increase of *btuB2* transcript abundance under starvation conditions suggests that BtuB2 expression represents a physiological adaptation to cobalt starvation.

Remarkably, the cobalt-dependent transcriptional regulation of *btuC* was lost in AFS-I-*btuB2*. In the presence of cobalt, the *btuC* transcript abundance was at least five-fold higher than that in the wild-type (**Figure 3C**, right graph, bar 1 vs. 5), paralleling the increase of *btuB1* (right graph, bar 5 vs. left graph, bar 3). In the absence of cobalt, the *btuC* transcript abundance was more than five-fold reduced when compared to the wild-type (right graph, bar 2 vs. 6) and below the expression level in the mutant strain when grown under normal growth conditions (right graph, bar 5 vs. 6). Thus, *btuC* expression resembles that of *alr4027* and *btuB2* in the wild-type and AFS-I-*btuB1,* while it qualitatively matches that of *btuB1* in AFS-I-*btuB2*. This is additional evidence that the transcription unit of *alr4027* extends beyond *btuC*. Moreover, as the reversal of the regulatory pattern of *btuC* in the *btuB2* mutant resembles the physiological regulation of *btuB1*, it indicates a relation of *btuB1* to the cluster of cobalamin-uptake related genes close to *btuB2*. On the other hand, in AFS-I-*btuB1,* the expression of *btuC* showed no alteration when compared to the wild-type under any treatment (**Figure 3C**, right graph, bar 1 vs. 3 and 2 vs. 4).

Based on the mentioned putative riboswitch upstream of *alr4027*, a hypothetical model of transcriptional regulation can be devised: Under cobalt-replete conditions, the cobalamin-dependent riboswitch is present in cobalamin-bound form destabilizing the transcript or repressing the transcription of a polycistronic mRNA that includes the riboswitch and the ORFs *alr4027-btuD*. Under these conditions, *btuB1* and a second mRNA including the ABC-transporter-related genes *alr4030*-*btuD* are transcribed from promoters upstream of *btuB1* and *alr4030*, respectively. Therefore, the genes for the cobalamin ABC-type transporter complex and *btuB1* are expressed at a moderate level while *btuB2* and *alr4027* are about 5±1-fold lower (**Figure 1E**, **Figure 3C**, *btuC* and *btuB1* vs. *btuB2*, wild-type). Cobalt-depletion leads to a reduction of cobalamin in cells and thus, a lower frequency of cobalamin-loaded riboswitches. Hence, the transcript level of the long *alr4027*-*btuD* (containing the riboswitch) mRNA is then stabilized and increases 200±100 times (**Figure 1E**, **Figure 3C**, *btuB2*, wild-type). A potential trans-inhibition of *btuB1* and the existence of the *alr4030-btuD* mRNA independent of the riboswitch are able to explain the other findings as well. As the induction of the long *alr4027*-*btuD* transcript increases the levels of the cobalamin free riboswitch, in the absence of cobalt *btuB1* is reduced by three-fold (to 0.3 ±0.1) in wild-type and I-*btuB2* when compared to conditions with cobalt. The short *alr4030-btuD* mRNAs is reduced by two-fold (to 0.5±0.1) in I-*btuB2* when compared to normal growth conditions (**Figure 3C**). Consistent with this notion, the short *alr4030-btuD* mRNAs is not reduced in wild-type as the levels are dominated by the long transcript.

In summary, *btuB2* is induced by cobalt starvation, but *btuB1* is mostly constitutively expressed. The cobalt-dependent increase of *btuC* depends on an element upstream of *btuB2*. Taking that into account as well as the regulatory patterns and the transcriptional start sites annotated [49] we suggest that the putative cobalamin-dependent riboswitch between *iacT* and *alr4027* regulates the transcript and protein abundance of the long single polycistronic mRNA including *btuB2*, *alr4030*, *btuF*, *btuC* and *btuD*. The transcription of this mRNA is presumably initiated at a start site within the ORF of *iacT*. This operon is Co-dependently transcribed via a mechanism presumably based in repression of transcription by the cobalamin-bound form of the riboswitch encoded upstream of *alr4027*.

### Impact of cobalt starvation on the intracellular metal reservoirs

**Figure 4:**
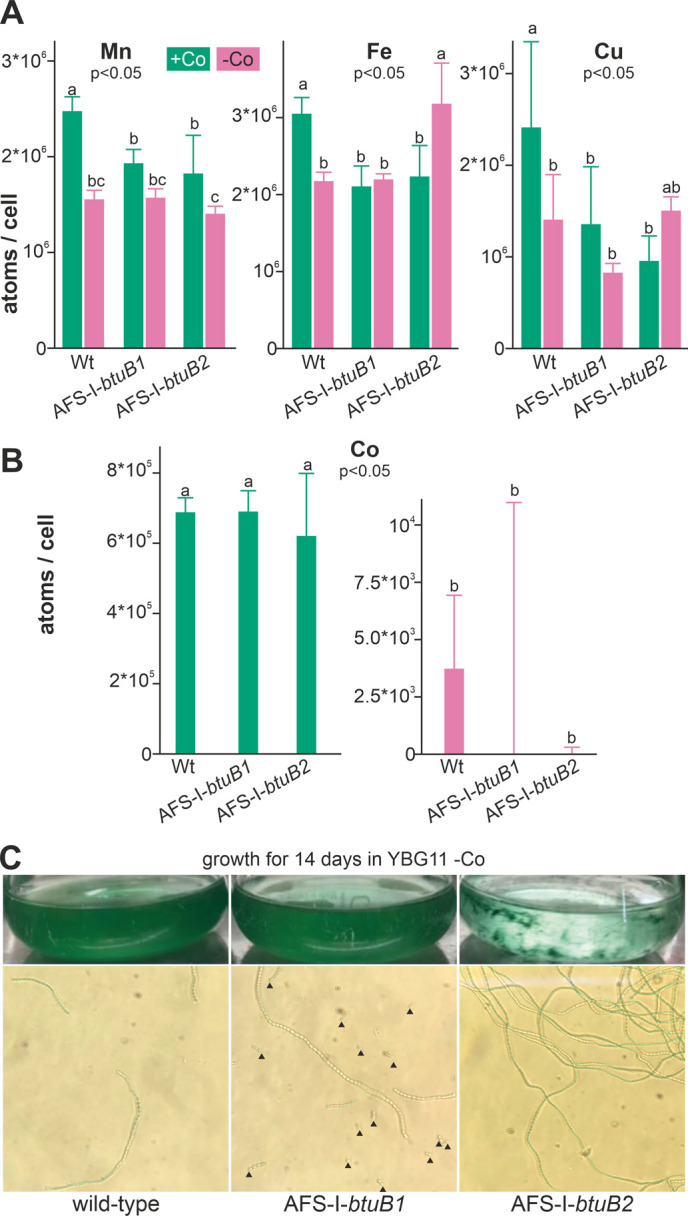
FIGURE 4: Influence of cobalt starvation on *Anabaena* and *btuB* mutants. **(A)** Wild-type *Anabaena*, AFS-I-*btuB1* and AFS-I-*btuB2* were grown for 5 days in YBG11 medium (+Co, green) or for 14 days in YBG11 medium without cobalt (-Co, magenta) followed by the determination of the intracellular manganese (left), iron (middle) or copper (right) content shown as atoms per cell. **(B)** The cobalt content of the strains used in A was determined, analyzed and represented as in A. In (A) and (B) the average of three independent experiments is shown; error bars indicate standard deviations. Statistical analysis on three independent replicas was performed as described in methods. The letters indicate the ranks based on statistical analysis performed using ANOVA (p<0.05). **(C)** Wild-type *Anabaena*, AFS-I-*btuB1* and AFS-I-*btuB2* were grown for 14 days in YBG11 medium without cobalt. Cultures were photographed (top) and filaments were visualized by light microscopy using a 40x objective (bottom). Black triangles indicate filament fragments or single cells.

To determine the impact of growth in cobalt deplete medium on intracellular availability of cobalt, cellular metal concentration was quantified by ICP-MS. Wild-type *Anabaena*, AFS-I-*btuB1* and AFS-I-*btuB2* were grown for five days in YBG11 medium or for two weeks in the absence of cobalt (YBG11-Co medium). The different incubation times were chosen to ensure that the cobalt content in the starved biomass was reduced to a minimum without depleting cobalt in the control cultures by prolonged incubation in exhausted medium. We did not observe any significant alteration of the cobalt content between the three strains grown in cobalt-replete medium (**Figure 4B**, left graph). This suggests that the basal expression of BtuB1 and/or BtuB2 protein(s) is sufficient for the required cobalt uptake under the conditions investigated. In contrast, the intracellular amount of cobalt was close to or below the detection limit in all samples, corresponding to at least 100-fold reduction, in all strains after two weeks of cobalt starvation (**Figure 4B**, right graph).

We also measured the cellular concentration of manganese, iron and copper to assess possible side effects of cobalt starvation. In the wild-type, the level of manganese, iron and copper was found to be significantly reduced under cobalt starvation (**Figure 4A**, bar 1 vs. 2) when compared to cobalt replete conditions. It is important to note that the potentially stronger exhaustion of the medium after cultivation for 14 days (Co-deplete cultures) opposed to five days (Co-replete cultures) may be responsible for some of the effects observed. In addition, Co-deplete and replete cultures might have been in different growth states. Thus, strains grown in the same medium were primarily compared. Indeed, compared to wild-type the levels of these three metals were reduced in the presence but not in the absence of cobalt (bar 3 and 4 vs. 2) in both mutants AFS-I-*btuB1* and AFS-I-*btuB1*. This shows that the content of iron, manganese and copper is generally affected in the two mutants, while with respect to iron and copper the reaction is mutation specific.

All mutants were viable after this starvation period, indicating that when grown in Co-replete medium *Anabaena* is able to store several orders of magnitude more cobalt than necessary for optimal growth. Visual inspection of the cultures did not show any difference between the wild-type and AFS-I-*btuB1*, while the biomass of AFS-I-*btuB2* showed cell aggregation (**Figure 4C**, top images). Microscopic analysis showed a higher degree of fragmentation of the filaments of AFS-I-*btuB1* when compared to the wild-type, whereas AFS-I-*btuB2* showed longer and less fragmented filaments (**Figure 4C**, bottom images). Thus, the two mutants showed a visually different phenotype. These phenotypes could be explained as follows. We observed an enhanced expression of *btuB1* in AFS-I-*btuB2* (**Figure 3C**). It could be considered an additional function for BtuB1 in adhesion as described for the TBDT encoded by *iha* in *E. coli* [51]. Such function would also be consistent with the fragmentation phenotype of AFS-I-*btuB1* due to the absence of the structurally relevant TBDT. However, based on our results this notion remains a hypothesis which needs to be explored in the future.

### Uptake of cobalamin by *Anabaena* increases with cobalt starvation status

**Figure 5:**
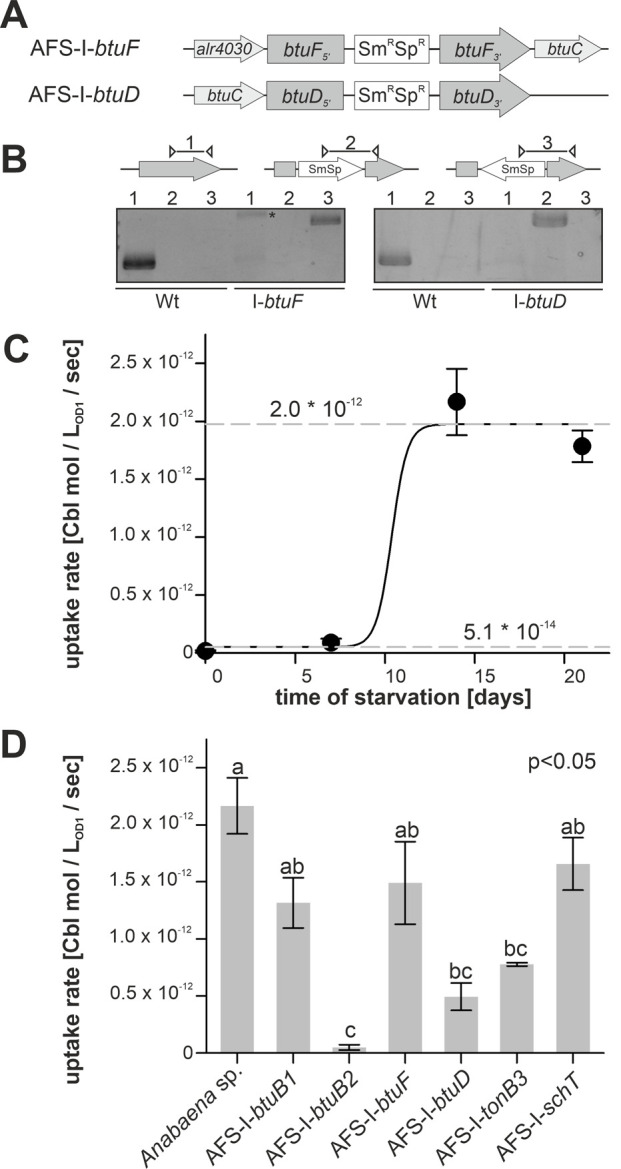
FIGURE 5: Cobalamin uptake by wild-type *Anabaena* and mutant strains. **(A)** Model of the strategy for genomic plasmid insertion to generate AFS-I-*btuF* and AFS-I-*btuD.* **(B)** Genomic DNA isolated from wild-type *Anabaena* and the indicated mutant strains was used for PCR with gene-specific primers (lane 1), a gene-specific primer and a plasmid-specific 3' (lane 2) or 5' primer (lane 3). The asterisk (*) indicates an unspecific PCR product. **(C)** Wild-type *Anabaena* was grown in YBG11 medium, followed by growth in YBG11 medium without cobalt for the indicated time. At these time points the uptake rate of cobalamin was determined as describe in methods. Data are shown as average of at least three independent experiments and standard deviation is shown as error bars. The line represents the least square fit result to equation 2. The dashed lines indicate the minimal and maximal uptake rates determined by this analysis. **(D)** The indicated *Anabaena* strains were grown in YBG11 medium, followed by growth in YBG11 medium without cobalt for 14 days. The uptake rate was then determined. Data are shown as average of the results of at least three independent experiments and standard deviation is shown as error bars. Statistical analysis on three independent replicas was performed as described in methods. Letters indicate the ANOVA results (p<0.05).

In addition to the generated mutants, plasmid-insertion mutants in *btuF* and *btuD* annotated as AFS-I-*btuF* and AFS-I-*btuD* were created by single recombination (**Figure 5A**). Both mutants were segregated, because the plasmid, but not the wild-type gene, could be detected (**Figure 5B**).

Cobalamin uptake velocity was determined with ^57^Co-cyanocobalamin. The conditions applied for uptake experiments are consistent with experimental conditions used for the analysis of cobalamin uptake by other bacteria [52]. The velocity was determined from linear regression of time-dependent measurements of radioactivity retention in ^57^Co-cyanocobalamin-exposed biomass. Wild-type cells grown in Co-replete medium without starvation showed marginal uptake rates of (5.1 ± 0.3) * 10^-14^ mol * L_OD=1_^-1^ * sec^-1^ (**Figure 5C**). Even after seven days of starvation a similarly low uptake rate was observed. From day 7 to day 14, the rate increased by two orders of magnitude to (1.97 ± 0.05) * 10^-12^ mol * L_OD=1_^-1^ * sec^-1^, which exhibited no further increase upon longer starvation (**Figure 5C**). The EC_50_ starvation time for the transition between low and high transport rate was 10 ± 1 days (**Figure 5C**). This observation is in remarkably good accordance with the observations on gene expression (**Figure 1–3**), which exhibited a higher *btuB2* expression under starvation. It appears reasonable to assume that the uptake rate increase results from the Co-dependent increase in *btuB2* expression upon transfer to cobalt-deprived medium.

Therefore, the mutant uptake rate was only analyzed after 14 days of starvation as no major effects of the mutations were expected for biomass grown in cobalt-replete conditions. To test whether the BtuB system is dependent on TonB3 [53] or other transporter such as SchT [18] can transport cobalamin, the respective mutants were included in the experiment. Mutants AFS-I-*btuB1*, AFS-I-*btuF* and AFS-I-*schT* exhibited the same uptake rate as the wild-type (**Figure 5D**). In contrast, the uptake rates observed for AFS-I-*tonB3* and AFS-I-*btuD* were about 4-fold reduced compared to the wild-type. The uptake by AFS-I-*btuB2* was significantly reduced by 50-fold when compared to that of the wild-type and indistinguishable to the uptake rate of the wild-type without cobalt starvation (**Figure 5D**). As demonstrated by the lowered uptake rate of AFS-I-*tonB3*, under the conditions of the experiment, TonB-dependent outer membrane (OM)-translocation is a rate-limiting step of cobalamin import in *Anabaena*. The reduced uptake rate in the mutant AFS-I-*btuD* confirms the hypothesis that the ABC-type import system encoded in the gene cluster functions in cobalamin uptake. Moreover, it demonstrates that the uptake rate observed for starved wild-type cells is also dependent on specific translocation through the plasma membrane. Interestingly, the even stronger uptake reduction observed for AFS-I-*btuB2* suggests that BtuB2 is more important for cobalamin uptake than the ABC-type transporter system composed of BtuCDF. Nevertheless, it must be considered that the Co-deprived expression of *btuC* was considerably reduced in AFS-I-*btuB2* (**Figure 3C**) and, thus, it could be assumed that the reduced cobalamin uptake of AFS-I-*btuB2* is partly caused by polar effects on downstream genes. However, plasmid insertion into *btuF* does not affect cobalamin uptake significantly (**Figure 5D**), which argues against an impact of polar effects. This might suggest that paralogs of the ABC-type transporter BtuCDF can facilitate cobalamin transport. Alternatively, it might be that as long as moderate amounts of the ABC-type transporter are present, the uptake rate depends on sequestration and OM-translocation by *btuB2*. This would also explain the stronger reduction found for AFS-I-*btuB2* compared to AFS-I-*btuD*. The idea that the ABC-type transporter is present in excess (under the chosen experimental conditions) is also congruent with the observation that the periplasmic binding protein BtuF appears to be redundant and that is widely accepted that ABC-type transporters can function with alternative substrate-binding proteins. Although the cobalt concentrations in the experiments were orders of magnitude above those expected in a natural habitat [54], the described findings are not in conflict with the concept that an increased cobalamin transport efficiency by starvation-induced expression of *btuC* and *btuD* as well as involvement of a periplasmic binding protein is essential for survival in a Co-deprived environment.

Remarkably, AFS-I-*btuB1* was not significantly affected in cobalamin uptake after cobalt starvation, while a regulatory interdependence of *btuB1* and *btuB2* was observed (**Figure 3C**). Nevertheless, considering both, gene expression and uptake observations, our findings are in agreement with the hypothesis that BtuB1 is a moderately- and constitutively-expressed cobalamin receptor able to cover the cells cobalamin demand under Co-replete conditions.

### Conclusion

As the central ion of the cofactor of methionine synthase [32–34] and by its ability to partially replace other micronutrients [40], cobalt is essential for cyanobacteria [35–39]. In agreement with this importance, components of the TonB-dependent cobalamin uptake system [15] and of a cobalamin synthesis pathway had been proposed for *Anabaena* by bioinformatics analysis [22].

In here, the involvement of cobalamin riboswitches in the regulation of the uptake-cluster has been approached (**Figure 1**), while for *all0456* and *all1781* the importance of this RNA element remains to be experimentally confirmed. We demonstrated that *alr4027*, *btuB2* and *btuD* are induced by cobalt starvation (**Figure 1–3**). There is evidence that *btuB2* is expressed as a polycistronic transcript spanning from *alr4027* to *btuD* under Co starvation. We conclude that this mRNA is most likely controlled by the cobalamin riboswitch encoded upstream of *alr4027*, which would parallel the mode of regulation in proteobacteria [45, 46].

In contrary to *btuB2*, *btuB1* is not induced by cobalt starvation or excess (**Figure 2**). However, *btuB1* transcript is about ten-fold higher abundant under control conditions when compared to *btuB2* (**Figure 3**; e.g. [55]). This might suggest that *btuB1* has a basal function while *btuB2* is required under starvation conditions. Consistent with an overlapping function, the transcript abundance of *btuB1* is enhanced in the mutant strain with disrupted *btuB2* under control and under starvation conditions when compared to the wild-type strain (**Figure 3**). Similarly, the *btuB2* transcript abundance is enhanced in the mutant strain with disrupted *btuB2* when compared to wild-type under control conditions, while the large increase of transcript abundance of *btuB2* under starvation conditions in wild-type is not further elevated in the mutant (**Figure 3**). This is consistent with a more important function of *btuB2* under stress conditions when compared to *btuB1*. Further support of the proposal of a complementary function of the two proteins comes from the observation that we were not able to generate a viable double insertion mutant disrupting both gene functions (not shown), while the two mutants AFS-I-*btuB1* and AFS-I-*btuB2* were fully segregated (**Figure 3**).

Further, we demonstrated the participation of BtuB2 and BtuD in cyanocobalamin uptake. The mutant strains with disrupted *btuB2* or *btuD* showed a reduction of cobalamin uptake after cobalt starvation (**Figure 5**). Together with the mode of transcriptional regulation, these results suggest that BtuB2 is required for the high-rate and high-affinity cobalamin uptake under cobalt-limiting conditions.

In turn, the mutant of *btuB1* showed a reduced uptake of cobalamin, which however was not significant (**Figure 5**). Considering the idea that BtuB1 serves as a transporter with slow rate and likely low affinity under normal growth conditions (**Figure 5**) one would not expect a similar contribution under stress as BtuB2, but under normal growth condition. Unfortunately, we did not measure the uptake rates of the mutants under normal growth conditions. In addition, the expression of *btuB2* was enhanced in AFS-I-*btuB1* grown in the presence of cobalt, which might in part complement for the absence of BtuB1 under normal growth conditions. Thus, future experiments need to target the functionality of BtuB1 to confirm its mode of action.

The functional diversification between BtuB1 and BtuB2, which we propose, is supported by the different phenotypes of the according mutants. AFS-I-*btuB1* shows a fragmentation phenotype (**Figure 4**), whereas elongated and aggregated filaments were observed for AFS-I-*btuB2* with enhanced *btuB1* transcript (**Figure 4**, **Figure 3**). This could for example be explained by a BtuB1 function in adhesion as described for other specialized TBDTs [51]. Although it has to be further explored, a functional diversification of both BtuB proteins that facilitate cobalamin uptake could enable *Anabaena* to regulate the speed and efficiency of cobalamin uptake and, thus, cobalt intoxication in an environment with high cobalt content or cobalt deficiency in an environment with low cobalt or cobalamin availability.

## MATERIAL AND METHODS

### Bacterial strains and growth conditions

Wild-type *Anabaena* sp. strain PCC 7120 (also known as *Nostoc* sp. strain PCC 7120 and *Nostoc* sp. ATCC 27893) and mutants derived from it (**Table 1**) were grown photoautotrophically at 30°C with 70 µmol photons m^-2^ s^-1^ in 50-ml Erlenmeyer flasks with constant shaking (100 rpm) or on plates. All flasks were treated with hydrochloric acid (10%) and extensively rinsed with ddH_2_O prior to use. Liquid YBG11 [56] or solid YBG11 containing 1% Bacto agar (Otto Nordwald GmbH, Hamburg, Germany) was used as standard medium. Mutant strains were grown in the presence of streptomycin and spectinomycin at 5 µg ml^-1^ each.

**Table 1.** Strains used in this study.

**Strain**	**Resistance**	**Genotype**	**Properties**	**Source**
*Anabaena* sp. PCC 7120			Wild type	C. P. Wolk
AFS-I-*btuB1*	Sm, Sp	*all3310*::pCSV3	Gene interruption by pCSV3 plasmid	[20]
AFS-I-*btuB2*	Sm, Sp	*alr4028*::pCSV3	Gene interruption by pCSV3 plasmid	[20]
AFS-I-*schT*	Sm, Sp	*alr0397*::pCSV3	Gene interruption by pCSV3 plasmid	[18]
AFS-I-*tonB3*	Sm, Sp	*all5036*::pCSV3	Gene interruption by pCSV3 plasmid	[53]
AFS-I-*btuF*	Sm, Sp	*alr4031*::pCSV3	Gene interruption by pCSV3 plasmid	This work
AFS-I-*btuD*	Sm, Sp	*alr4033*::pCSV3	Gene interruption by pCSV3 plasmid	This work
AFS-I-P*btuB1*	Sm, Sp	P*all3310*-*gfp*	N-terminal GFP fusion	This work
AFS-I-P*btuB2*	Sm, Sp	P*alr4028*-*gfp*	N-terminal GFP fusion	This work
AFS-I-P*iacT*	Sm, Sp	P*all4026*-*gfp*	N-terminal GFP fusion	This work
AFS-I-P*alr4027*	Sm, Sp	P*alr4027*-*gfp*	N-terminal GFP fusion	This work

AFS: Anabaena Frankfurt Schleiff; Sm: streptomycin; Sp: spectinomycin

For cobalt starvation medium, Co(NO_3_)_2_ was omitted from the medium (YBG11-Co). For starvation experiments, both Co-deplete cultures and Co-replete controls were inoculated from biomass that was pre-starved in YBG11-Co for two weeks, unless stated otherwise. Pre-starvation of wild-type and mutant cultures was always carried out in parallel with identical treatment. Pre-starvation was obtained by inoculation at OD_750nm_ = 0.02 and growth for seven days, followed by harvesting, washing with YBG11-Co medium and reinoculation at OD_750nm_ = 0.02 for growth for seven additional days in YBG11-Co medium. For visual inspection, cultures were photographed and cells analyzed using an Olympus CKX41 microscope with a 40x objective.

### DNA and mRNA isolation and analysis

*G*enomic DNA was isolated from 50-ml cultures as described [57]. RNA was isolated from strains grown in YBG11 medium for seven days or in cobalt-depleted medium (YBG11-Co) for two weeks as described above. RNA was extracted with TRIzol (Thermo Fisher Scientific, Waltham, Massachusetts, USA). RNA isolation and DNaseI treatment were performed as described [50]. Synthesis of cDNA was done with RevertAid Reverse transcriptase from Thermo Fisher Scientific. qPCR was performed with a StepOnePlus Cycler (Thermo Fisher Scientific) and with PowerUp SYBR Green Master Mix (Applied Biosystems, Waltham, Massachusetts, USA) using gene specific oligonucleotides (**Table 2**). The Ct value of the gene of interest was normalized to the Ct value of *rnpB* (Δct, [58]). PCR efficiencies were calculated by fitting the amplification curves (**Table 3**) using the equation developed by Spiess *et al.* [59]. Purity of the PCR product was checked by recording of DNA melting curves.

**Table 2.** Oligonucleotides used in this study.

**Purpose**	**Name**	**Sequence**
Cloning	BtuB1-PGFP-fw	ATCGATGCTGATGAGAAGTTGTTTTGG
	BtuB1-PGFP-rv	GATATCTCTCACTTCGTCTCACACCAAG
	BtuB2-PGFP-fw	ATCGATATTTGTCTGCACGACCTGC
	BtuB2-PGFP-rv	GATATCCATTGGTAATTGAAGCCACCTG
	IacT-PGFP-fw	ATCGATGCAGGTAGGCGTAAAGTTAC
	IacT-PGFP-rv	GATATCCACACCACGAGACTGAGAAC
	alr4027-PGFP-fw	ATCGATAGACGAGTGTAACAGCAATACCC
	alr4027-PGFP-rv	GATATCCATTGATTTTCAGGCGTTGC
	SR-alr4031-fw	GATCAGATCTCACCCACGCCAGAGGTTTG
	SR-alr4031-rv	GATCAGATCTTCCCAATGGCGTATCTTCC
	SR-alr4033-fw	GATCAGATCTTGCGGGTAAGTCTACATTACTGC
	SR-alr4033-rv	GATCAGATCTACCAAATCTTCAGGTGCGCC
Segregation analysis	GFP-4	CAAGAATTGGGACAACTCC
	pCSV3-R	CTGATGCCGCATAGTTAAGCC
	BtuB1-S-fw	CAAGGTGCTACCAGTGTCGC
	BtuB1-S-rv	GTGTGATGTCGTAACGTAGCCC
	BtuB2-S-fw	CAGCATGATAGCCACAGAAGTTC
	BtuB2-S-rv	CCTTAACGCCTTATCAACCTCC
	alr4031-S-fw	TTCCTCATAGCCACCCTCATCATCGC
	alr4031-S-rv	TCGCTACGTAACTATTCCCACCAGGC
	alr4033-S-fw	TCGGTGTGTTGCATCAGATATTTCGG
	alr4033-S-rv	AGTCCACACAGATGCGATACCTTCACC
	BtuB1-PGFP-S-fw	CCTGCACTGACGCTGAATTC
	BtuB1-PGFP-S-rv	GACTGATTAATTGAGGCTCCCC
	BtuB2-PGFP-S-fw	CTCCTCGTTGCCTTAGTTTCTC
	BtuB2-PGFP-S-rv	GAACCGCGAATAAATTGACC
	IacT-PGFP-S-fw	CCAGAACCGAGTTGATATTGAAAA
	IacT-PGFP-S-rv	GCCTTCTTGAAGAGTTTCTGGTG
	alr4027-PGFP-fw	CTCCTGGGATTTCCGTGTC
	alr4027-PGFP-rv	GCTTCTGGACGTTCTGACC
RT-PCR	rnpB-RT-fw	AGGGAGAGAGTAGGCGTTGG
	rnpB-RT-rv	GGGTTTACCGAGCCAGTACC
	BtuB1-RT-fw	GAATTGTGGCTGGAAGATGG
	BtuB1-RT-rv	GACGGGACGAAATCGGTAG
	BtuB2-RT-fw	GCAATCACATCTCAAAACCG
	BtuB2-RT-rv	GGTGATGTAAATGAGCCATTTAC
	IacT-RT-fw	CTGGATTGGTGGTAGCTTCC
	IacT-RT-rv	CACAGTCAGCGCCAACC
	alr4027-RT-fw	GGCGAAAGTGGTGGACAG
	alr4027-RT-rv	CTTAGGGGTGGAATTTTGGC
qPCR	rnpB-qRT-fw	GTAGGCGTTGGCGGTTG
	rnpB-qRT-rv	CACTGGACGTTATCCAGC
	BtuB1-qRT-fw	GGGCTACGTTACGACATCAC
	BtuB1-qRT-rv	TGCTTCCCAACCATGAACTG
	BtuB2-qRT-fw	GAATTTAGGAGTGCGGCAAG
	BtuB2-qRT-rv	GTGCGGAGTGTAGTTGAATC
	alr4032-qRT-fw	GTTGTGCTGACAGCAAGTG
	alr4032-qRT-rv	CCGAGACTTGCTGCTATAAC
	alr4027-qRT-fw	CAATGCGCCACTCAATAC
	alr4027-qRT-rv	CTTAGGGGTGGAATTTTGG

**Table 3.** pCR Efficiency for the different oligonucleotide pairs.

**Gene**	**Absolut efficiency**	**Relative Efficiency (%)**
*btuB1 (all3310)*	1.633 ± 0.007	81.6 ± 0.3
*alr4027*	1.649 ± 0.007	82.5 ± 0.3
*btuB2 (alr4028-alr4029)*	1.63 ± 0.01	81.5 ± 0.5
*btuC (alr4032)*	1.699 ± 0.004	84.9 ± 0.2
*rnpB*	1.681 ± 0.005	84.0 ± 0.3

### Genetic procedures

*Escherichia coli* DH5α, HB101 and ED8654 were used for plasmid constructions as well as conjugations into *Anabaena*. Conjugal transfer of plasmids into *Anabaena* was performed as described [53, 60].

Generation of mutant strains AFS-I-*btuB1*, AFS-I-*btuB2,* AFS-I-*schT* and AFS-I-*tonB3* has been described elsewhere [18, 20, 53]. To generate the strains AFS-I-P_*btuB1*_, AFS-I-P_*btuB2*_, AFS-I-P_*alr4027*_ and AFS-I-P_*iacT*_, 703 bp, 638 bp, 1119 bp and 1319 bp of the 5'UTR region of *btuB1*, *btuB2*, *alr4027* and *iacT*, respectively, including the start codon were amplified by PCR on genomic *Anabaena* DNA (**Table 2**). Fragments were cloned into the pGEM-T Easy vector (Promega GmbH, Walldorf, Germany), sequenced and excised by ClaI/EcoRV cleavage for cloning into pCSEL21, which contains the *gfp* ORF (). Digestion with EcoRI resulted in the final fusion fragment, which was ligated to pCSV3. Conjugation into *Anabaena* was performed as described [53, 60]. PCR confirmed the incorporation of the fragments into the *Anabaena* genome.

Mutants AFS-I-*btuF* and AFS-I-*btuD* were generated by amplifying an internal fragment from *Anabaena* genomic DNA. Restriction was performed with a *Bam*HI restriction site and the product was cloned into the pCSV3 plasmid [61]. The plasmid was transferred to *Anabaena* by conjugation [60].

**Table 4.** Plasmids used in this study.

**Plasmid**	**Resistance**	**Insert**	**Purpose**	**Reference**
pGEM-T	Amp		Cloning	
pCSV3	Sp^R^/Sm^R^		Cloning	[61]
pCSEL21	Amp^R^	*gfp-mut2*		[65]
pCSV3-btuB1	Sp^R^/Sm^R^	Internal fragment of *all3310*	Generation of single-recombinant mutants	[20]
pCSV3-btuB2	Sp^R^/Sm^R^	Internal fragment of *alr4028*		[20]
pCSV3-alr4031	Sp^R^/Sm^R^	Internal fragment of *alr4031*		This work
pCSV3-alr4033	Sp^R^/Sm^R^	Internal fragment of *alr4033*		This work
pCSEL21-btuB1	Sp^R^/Sm^R^	Internal fragment of *all3310*	Generation of promoter-*gfp* fusions	This work
pCSEL21-BtuB2	Sp^R^/Sm^R^	Internal fragment of *alr4028*		This work
pCSEL21-iacT	Sp^R^/Sm^R^	Internal fragment of *all4026*		This work
pCSEL21-alr4027	Sp^R^/Sm^R^	Internal fragment of *alr4027*		This work

Sp: spectinomycin; Sm: streptomycin; Amp: ampicillin

### Measurement of GFP fluorescence

GFP fluorescence measurements were performed using a Tecan Spark 10M plate reader (Tecan Trading AG, Männedorf, Switzerland). The excitation wavelength was 488 nm, GFP was measured at 533 nm and the optical density was determined at 750 nm. For reference a fluorescein solution in a concentration of 1 ng/ml was used. Each sample had a volume of 200 µl and was measured in a black 96-well microplate with a clear bottom (Greiner Bio-One GmbH, Frickenhausen, Germany). The GFP signal was normalized to the optical density and to the fluorescein reference. Lastly, the fluorescence signal of the mutant was normalized to that of the wild-type.

### Inductively coupled plasma mass spectrometry (ICP-MS)

Total cellular metal concentrations were determined as described [62, 63]. In short: cultures were grown for five days either in YBG11 medium or for two weeks in YBG11-Co medium (with reinoculation after seven days as described in “Bacterial strains and growth conditions”). Cells were then harvested and washed twice with a buffer containing 20 mM 2-(N-morpholino)ethanesulfonic acid at pH 5 and 10 mM ethylenediaminetetraacetic acid. Samples were resuspended with 5 ml of double distilled water and OD_750nm_ was measured. 1 ml of the resulting cell suspension was digested overnight at 120°C in 7 M HNO_3_ and dissolved in 5% HNO_3_ for ICP-MS measurement.

### Short-term uptake of ^57^Co-cyanocobalamin

For uptake measurements cultures were pre-starved for 14 days as described earlier, unless otherwise indicated. Prior to uptake experiments, *Anabaena* cultures were washed three times with YBG11-Co medium. Uptake was measured in 13 ml of cultures of OD_750nm_ = 0.2. 20 µl of ^57^Co-Cyanocobalamin (stock solution of 0.5 µCi, MP Biomedicals GmbH, Eschwege, Germany) was mixed with 13.6 µl of cyanocobalamin (stock solution 50 nM, Sigma-Aldrich, St. Louis, Missouri, USA) to adjust the final concentration to 92 pM. Cells were incubated in 50-ml falcon tubes in a water bath at 30°C under constant shaking in the dark. 0.5, 4, 8 and 16 minutes after the addition of cyanocobalamin, 2 ml of cell culture were filtered on a hydrophilic membrane (25 mm, 0.45 µm pore size, Merck, Darmstadt, Germany). After filtration, cells were washed three times with 1 ml of washing buffer (1 mM cyanocobalamin, 2 mM NaHCO_3_, 20 µM EDTA). Filter digestion and dissolution was previously described [64]. 10 ml of Aquasafe 300+ scintillation liquid (Zinsser Analytic, Frankfurt, Germany) was added and the ^57^Co-cyanocobalamin determination was done with a Hidex 300 SL liquid scintillation counter (Hidex Deutschland Vertrieb GmbH, Mainz, Germany).

### Quantification of cobalamin uptake

For each individual uptake experiment the initial rate was determined by


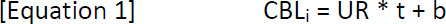



where CBL_i_ is the cyanocobalamin incorporated [mol/L_OD=1_], UR the uptake rate [mol/sec/L_OD=1_], t the time of incubation [sec] and b the background binding to cellular surface at time point zero [mol/L_OD=1_]. The rates determined for the individual experiments were used to calculate the mean and the standard deviation. The starvation-dependent behavior (Figure 4A) was analyzed with a sigmoidal curve to determine the starvation level


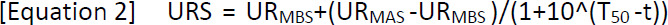



where URS stands for the uptake rate at a given time of starvation [mol/sec/L_OD=1_], UR_MBS_ the minimal uptake rate before starvation, UR_MAS_ the maximal uptake rate after starvation, T_50_ is the time of starvation with an uptake rate increase of 50% and t is the time of starvation [days].

### Statistical analysis

The statistical analysis of the metal content or the transport rates was performed with Sigma Plot (SPSS) by ANOVA (One Way, Normality Test: Shapiro-Wilk and Equal Variance Test: Brown-Forsythe (passed)). The classification was performed with p<0.05.

## Abbreviations:

DMB: – 5,6-dimethyl-benzimidazole,
GFP: – green fluorescent protein,
OM: – outer membrane,
TBDT: – TonB-dependent transporter.
